# Memoirs on Poisoning

**Published:** 1841-01

**Authors:** 


### Art. II.

*Mémoires sur l'Empoisonnement.* Par M. Orfila. Mémoires de l'Académie Royale de Médecine. Tome viii.—*Paris*, 1840. 4to.

*Memoirs on Poisoning.* By M. Orfila. From the Memoirs of the Royal Academy of Medicine. Vol. viii.—*Paris*, 1840. 4to.

The Memoirs of M. Orfila present so much of what is new and interesting in relation to toxicology, that we have thought it our duty to extract the article from the Memoirs of the Academy, and treat it as a separate work. These Essays on Poisoning occupy about 200 pages, quarto, of the volume, and are divided into five chapters on Arsenic, one on Tartarized Antimony, and one on Copper. The main object of the author is to make public certain new processes which he has discovered for the detection of these poisons, as they exist in the bodies of individuals who have died under their operation.

The chapters on Arsenic are undoubtedly the most important; they abound with observations susceptible of the most extensive application for good or for evil in the practice of medical jurisprudence; they require cool and deliberate examination, and, above all, it is necessary for a reader to guard himself against being misled by the enthusiasm of the author. Already a case of remarkable interest has occurred in France, in which a practical application of M. Orfila's new views has been made; we allude to the recent trial of Madame Laffarge for poisoning her husband with arsenic. In this case, the chemical processes commonly employed and recommended for the detection of arsenic entirely failed, but M. Orfila satisfied himself that arsenic was present in various organs of the body of the deceased, although it had not been detected in the stomach. His evidence seems to have considerably influenced the jury, and to have led them to pronounce a verdict of guilty. We think we have said enough to show that these alleged discoveries cannot, if well founded, be too widely circulated; and it must rest with the profession to determine whether they are worthy of that confidence, in a judicial light, which the author so earnestly claims for them.

The facts upon which M. Orfila chiefly dwells, and which he considers are fully established by his experiments are: 1, the absorption of arsenic into the blood when applied externally or taken internally; 2, the presence of that poison in the heart, brain, lungs, and other organs of individuals who have died from its effects; 3, a new method of separating arsenic from organic matter, and of detecting it by Marsh's apparatus; 4, the presence of arsenic as a *natural* constituent of human bone, and its probable presence as such in the muscular system; 5, its non-existence in the soft organs, except in cases where the individual has been the subject of poison; 6, its presence in the soil of cemeteries, rich in the detritus of human bone.

It had been long suspected by toxicologists that arsenic entered into the blood by absorption; but this suspicion was founded on physiological inference, and not on any ascertained facts. In nearly all modern works on toxicology, the reasons why arsenic could not be detected in that liquid are assigned, and the search for it was regarded as hopeless. These reasons were chiefly, that the poison itself existed in but small quantity, and that but a very small portion of blood could be operated on at once. M. Orfila has overcome both these difficulties, and a new argument in favour of the action of poisons by absorption has been thereby added to those which previously existed. In the first memoir there are given the details of thirty-two experiments, chiefly performed on animals, for the purpose of showing that arsenic actually enters into the blood. In some of these the poison was inclosed in small bags, which were placed in wounds made in various parts of the body; in others the poison was introduced into the stomach, the œsophagus being afterwards tied, and the blood of the animal at a certain period was abstracted and examined. The poison was detected by the use of Marsh's apparatus in all cases where a sufficiency of blood was operated on, or a sufficient quantity of the soft parts taken. In one case the blood was removed from the abdominal aorta of the living animal one hour and twenty-five minutes after the poison had been placed within the stomach, and the œsophagus tied; the blood of this animal yielded traces of arsenic: "the brain contained a little, the lungs rather more, the heart and kidneys still more, but the liver and spleen yielded the largest quantity." (Exp. 16, p. 386.)

In the course of these experiments, the author attempted to determine how much of the poison was required to destroy life. Thus, he dried and weighed the small bag containing the poison before he introduced it into the wound, and after the death of the animal he again dried and weighed it. There was a trifling loss of weight. (Exp. 2, p. 378.) As the author does not insist upon the practical application of these results, we shall not criticise the experiments further than to observe that, in this point of view, they are to our minds wholly inconclusive. The necessary admixture of the blood of the wound with the bag and its contents must render any inference, founded on a loss of weight before and after the experiment, fallacious. This alleged loss of weight in the quantity of poison employed seems to have been mainly instrumental in causing him to extend his researches, and to apply his processes for its detection in the soft organs of the body.

His first efforts to detect the poison were not very successful, owing to his having examined too small a quantity of the soft parts. By taking several organs and acting on them by one process, the evidence of the presence of poison became conclusive. Within a very short time after the announcement of Marsh's process for detecting arsenic, and before it could have become known in France, we employed the apparatus in order to detect arsenic in the blood of two animals which had been poisoned by it, but the result was unsatisfactory; no evidence of the presence of arsenic could be obtained. Since the publication of Orfila's experiments we are satisfied that the cause of failure was to be ascribed to two circumstances, both of which were an embarrassment to him in the outset: 1st, that too small a quantity of blood was taken; and 2d, that the minute portion present was probably concealed and protected from the action of the chemical reagents by the organic matter mixed with it. The last fact is interesting; it has hitherto escaped the attention of toxicologists, and we cannot but conclude with the author, "that arsenic may have been present in many cases where medical witnesses have pronounced it to be absent." (p. 422.) Orfila has removed this difficulty by insisting on the preliminary process of incineration with nitrate of potash, thereby changing the arsenic into a fixed arseniate, or what he considers to be an improvement on his original plan, digesting the animal matter containing arsenic in nitric acid, and thereby oxidizing and destroying it. We shall advert to the details of this process hereafter. One other point requires to be noticed. If, on passing sulphuretted hydrogen gas into a suspected liquid, there should be no production of sesquisulphuret of arsenic, indicated by a change of colour, it would be affirmed by most toxicologists that arsenic was not present. Orfila contends that arsenic may be present in such a case, and that it may also exist in a liquid in which all the sulphuret has been thrown down by the gas. He has proved this by dissolving a quantity of nitrate of potash in the liquid, evaporating to dryness, incinerating and introducing the saline residue, previously washed with sulphuric acid, into the hydrogen apparatus. (p. 396.) It would be useless to enter into further particulars at present, since, in the third memoir, the author substitutes another process for that which he recommends in the first.

A large portion of this memoir is devoted to a description of Marsh's apparatus, and the precautions to be observed in using it. We agree with him, that the plan proposed by the inventor for detecting arsenic in organic liquids is complex and inconvenient of application; not to mention that the detection of the poison under these circumstances must be very uncertain. Thus, Mr. Marsh advises that the whole organic liquid, to the amount of three or four pints or more, should be placed in a vessel and hydrogen generated in the midst of it. Orfila found this method so unsatisfactory that he soon abandoned it. The chief difficulty to the disengagement of arsenuretted hydrogen he found to exist in the large quantity of froth produced by the presence of organic matter. He, in vain, endeavoured to prevent this by the use of a stratum of oil, as recommended by Mr. Marsh. Objections have been repeatedly made to the use of the apparatus on account of the froth thus formed, but we have found that the production of this is easily avoided by pouring a small quantity of pure alcohol on the surface of the liquid. In no part of Orfila's treatise is this simple remedy adverted to, although it is actually mentioned by Mr. Marsh in his original communication to the Society of Arts. Granting that the froth is got rid of, there is, as Orfila contends, an insuperable objection to operating on so large a quantity of organic liquid, in which there may be only traces of arsenic; and it is clear, if but a small quantity be taken, as in the employment of Marsh's bent tube, we may be deceived in our judgment respecting the presence of poison.

The entire destruction of the organic matter, the fixation of the arsenic during this process, and the consequent reduction in the bulk of the materials, have enabled Orfila to detect arsenic in the minutest traces, in a small apparatus, and without the production of any froth.

The apparatus of Orfila consists simply in a bottle, with a bent glass tube drawn to a point. It is undoubtedly susceptible of improvement: there ought to be a stopcock attached, to prevent the escape of gas. If any froth should form, alcohol may be used—not oil, since this is not merely inconvenient, but it is apt to absorb and retain a portion of the arsenuretted hydrogen produced. The only inconvenience resulting in such a case from the employment of alcohol is, that it is apt to precipitate the sulphate of zinc produced in the process; an effect, however, which never takes place, unless the sulphuric acid is nearly saturated with oxide of zinc.

The method of obtaining a metallic sublimate or a deposit of arsenious acid must be so well known to our readers, that we need not follow our author in his description. There is here nothing new. Orfila places such implicit confidence in the results, that he considers any other process for the detection of arsenic, mixed with organic matter, superfluous. It is at this point, we think, he carries his views too far; and, as this involves a question of the highest importance in legal medicine, it will be necessary to examine the grounds on which he rests his exclusive opinions. The question in the most simple form is this: Supposing that there was no evidence of the presence of arsenic in an organic liquid on the application of the usual tests, while the hydrogen apparatus gave a brown stain on glass, is this a satisfactory proof on a criminal trial of the existence of the poison? According to Orfila, it would be deemed so under certain limitations; and we shall now describe the characters which he assigns to true arsenical stains, as well as the means which he proposes to distinguish them from others which resemble them. We may premise that, very soon after the announcement of Marsh's discovery, Mr. Lewis Thompson ascertained that antimony was liable to be taken up by a current of hydrogen, and to become deposited like arsenic on cool surfaces exposed to the flame.

"1. The stains of *arsenic* are of a brownish yellow colour, and resplendent; when the arsenic is in large quantity they are of a brown black; when the proportion is very small, or the poison is mixed with organic matter, they are of a canary yellow colour. The stains of *antimony* are of a deeper colour, blue and brilliant when thick, but of a yellow brown when the layer of metal is very thin. Neither the stains of arsenic nor those of antimony are volatile at common temperature; they are not deliquescent, nor do they redden litmus paper. 2. An *arsenical* stain, of whatever thickness, is entirely volatilized in from half a minute to a minute, when exposed to the flame of hydrogen gas, as in the common philosophical lamp. The antimonial stain, on the contrary, even when thin, is not volatilized on exposure to this flame until after the lapse of five or six minutes. The stain spreads, becomes lighter coloured, white oxide of antimony is formed and volatilized, but a grayish yellow spot always remains. 3. The stains of both metals are easily dissolved by a few drops of strong nitric acid; and, on evaporating to expel the excess of acid, a white or whitish yellow residue is obtained from arsenic (a mixture of arsenious and arsenic acids), and a yellowish residue with antimony (yellow oxide). If a drop of a solution of neutral nitrate of silver be poured on the *cooled* residues, the oxide of antimony undergoes no change, while the arsenical residue is converted into a brick-red arseniate, mixed with yellow arsenite of silver. If the brick-red arseniate be treated with a drop of ammonia, the red colour comes out clearer; the oxide of antimony, on the other hand, mixed with nitrate of silver, becomes blackened as soon as it is touched by a drop of that alkali. 5. If, after having used the last test, we can procure more metallic stains, these may be dissolved in diluted nitric acid:—the solution evaporated to dryness, and the white residue obtained dissolved in boiling water. The aqueous solution thus procured, acidulated with one or two drops of muriatic acid, may be treated with sulphuretted hydrogen; if the stains be *arsenical*, yellow sulphuret of arsenic will result, which will be particularly developed on boiling the liquid. Antimonial stains thus treated, yield with sulphuretted hydrogen an orange-red precipitate." (p. 405.)

M. Orfila thinks that a practised eye would easily distinguish by the colour the flame of arsenuretted from that of antimoniuretted hydrogen. He recommends that, on the performance of these experiments, the two gases should be produced and burnt in distinct apparatuses. The flames in each case give off a white vapour if the respective metals be in large proportion, but there is no white vapour when the quantity is small. (p. 408.)

The author declares that it is not necessary to procure the five characters above mentioned in order to affirm that the stains are of an arsenical nature.

"The stains which present the first three characters, and one of the two others, should be pronounced arsenical, and, à fortiori, when all the five characters exist, there can be no doubt of their being formed of arsenic. Strictly speaking, one who is much experienced in researches of this kind might decide on the presence of arsenic when he procured the *first three characters only,* especially when the stains have resulted from acting on the incinerated residue of the alimentary canal, liver, spleen, kidneys, lungs, or heart; for there is no substance which can yield stains possessing these three characters, when, after having been mixed with the organic tissues, it has undergone the carbonizing (?) action of concentrated nitric acid." (p. 408.)

Thus, then, we learn that M. Orfila would rely upon the appearance of the stain, its entire volatilization when exposed to a flame of hydrogen, and the colour of the residue left on evaporating a solution of the stain in concentrated nitric acid. We must remember that we are here called on to form an opinion, in which we assume there is no other evidence to assist us; and this opinion may turn the scale in the minds of a jury against an accused party. How cautious, then, should we be of grounding our judgment upon circumstances open to the least doubt or suspicion! We hope to show that, in these extreme instances in which ordinary chemical analysis fails, the criteria proposed by M. Orfila are not safe, and that they are wholly unfit for the guidance of a medical jurist in a case involving the question of the life or death of an accused party. We should hesitate to express ourselves thus strongly, were it not that, on more than one occasion recently, these views have been laid before the public, and have had considerable influence on the verdicts of juries. The reputation which our author has so deservedly acquired as a toxicologist, has led to his opinions being received, both by the public as well as many professional men, without that rigorous examination which opinions on such delicate subjects, and involving so serious an alternative to accused parties, would have met with had they emanated from men of less repute. After all, the question of poisoning in criminal jurisprudence is one of fact: the reputation of the witness cannot affect the facts on which his evidence is founded, while it may dispose us to receive them without sufficiently examining how far they warrant the opinion which he expresses. If any man could thus lead the judgment of others in those questions, it is the author of these Memoirs, and we therefore hold it to be our duty to determine from his own statements whether he is warranted to do so in the case before us.

In the first place, with regard to the appearance of the stains. Antimony never presents, according to our observation, the decided metallic lustre which often results from arsenic, although looking at the antimonial stain from the back of the glass, a metallic glimmering is apparent.

When, as it often happens, the arsenical stain is free from lustre, the appearance alone cannot distinguish it from antimony. From this it follows, that the antimonial stain may be often mistaken for the arsenical; the same black incrustation, destitute of metallic lustre, may exist in the case of either metal. We do not deny that those who have experimented much on this subject may be able to form a conjecture from the appearance; thus we have observed when the stains are taken on glass, and we view them by transmitted light both are opaque in the centre; but the arsenical stain is *brown* at the border, while the antimonial, is of deep gray *black*. Still legal evidence must not be based upon conjectures; and that there is strong room for doubt, is proved by the author himself advising us to search for other characters before we decide. Now what are these other characters? the rapid volatilization of the arsenical stain by heat is a weak criterion, for we have found that the antimonial stain is entirely volatile, and the difference is only one of time; the deposit in the latter case being driven off more slowly than in the former; indeed, if the flame should by any means meet the layer of antimony, it is soon mechanically dissipated by the current, while, if the arsenical stain is collected on a thick plate of glass, or held at a distance from the flame, it is very slow in disappearing. But allowing with Orfila that there is the difference of time mentioned by him in the two cases, is the bare fact of a sublimate being volatilized in one minute or in five minutes, sufficient to justify a medical witness in pronouncing an opinion on the presence of arsenic, in a question of life and death? According to our view, it is not: no man is justified in acting upon such niceties, and assuredly no evidence founded upon a fact of this kind would have any weight in an English court of justice. 3. The solution in nitric acid, with the colour of the residue obtained on evaporation, furnishes no safer ground of distinction, when such minute quantities are the subject of experiment. We might make the same remark with regard to all the other alleged distinctions, pointed out by the author. Perhaps the best of these is the *fifth;* when the crust first dissolved in dilute nitric acid is evaporated, again dissolved in water, and treated with sulphuretted hydrogen. A yellow sesquisulphuret results with arsenic, an orange red, with antimony. We may here remark, however, that if such a difference of colour can be observed in operating on the crust, there must be in general enough arsenic or antimony present, to allow of the free application of other tests, and to indicate the nature of the metal beyond all dispute. Our objections apply to cases in which we are obliged, if we form any opinion at all, to decide from the hydrogen test alone. How is it possible to obtain evidence, such as that which we are advised to seek for, from stains which may not weigh the thousandth of a grain? or who is always to distinguish in these minute pellicles of liquid, the colour produced by sulphuretted hydrogen in the case of antimony, from that formed when arsenic is present? The author obviously rests his decision chiefly on the first three criteria; but individually, we have proved these to be liable to fallacy, nor do we see how, when taken collectively, they can acquire such force as to become the basis of a sound medical opinion. Besides, in giving these statements to the world, it is not considered that the experiments will probably be performed by many, who, although tolerably versed in chemistry, may not come up to the standard of experience indicated by M. Orfila. In such hands the most dangerous mistakes may arise, if too great a reliance be placed upon criteria, which, while they are themselves weak, require considerable nicety in their application.

But does it follow that we are without any means for distinguishing the arsenical from the antimonial crust? certainly not; we intend by these remarks to show that we must not rely on our tests, where only minute traces of the suspected substance can be procured. The chemical differences pointed out by M. Orfila are applicable with safety where the metallic sublimate is procured in moderate quantity; but after all we have found it better to test the liquid from which the sublimate is derived, than the sublimate itself; a slip of bibulous paper dipped into any organic liquid containing arsenic or antimony will clearly indicate the nature of the metal present, by the colour assumed on exposing it to a current of sulphuretted hydrogen gas, with the action of ammonia on the coloured sulphuret formed. If no change of colour take place, under these circumstances, we much doubt whether, in the event of a sublimate being procured by the hydrogen apparatus, the quantity would not be too small to allow of our forming a safe opinion.

We have thus thought it right to dwell on this part of M. Orfila's analytical process; because most of the new views which he has produced in these memoirs are materially affected by the means which he employs to identify the arsenical stains.

Antimony is not the only substance liable to give rise to doubt. The author candidly states, that he has observed stains to result even from organic matter only. In some cases, where no arsenic was present, brilliant stains of a brown colour were produced, which differed from those of arsenic in being less volatile, and having none of the chemical characters of that metal. He has found stains to result from the presence of phosphorus, iodine, bromine, sulphur, selenium, and tellurium, (p. 410.) It is true that chemical differences exist in all these cases; but in a practical light, we cannot escape the inference, that the mere production of a stain in a suspected case is insufficient; and that the application of tests to this stain, where it is small, and the experimentalist not well versed in chemistry, must always leave a doubt on the mind as to its real nature.

But the author, with his acknowledged skill, candidly admits that he has found himself at a loss to determine the nature of these metalloidal stains in certain cases. The same difficulty may easily occur to others, who rely too exclusively upon the hydrogen test. We shall therefore transcribe his statement:

"There are other stains more important than those just adverted to, because they are often produced and may be sometimes confounded with those which are really of an arsenical nature. They are particularly observed to result from the solution of the incinerated residue of muscles, which have been acted on by strong nitric acid. They assume different characters; they are white, opaque, immediately volatile in the flame of hydrogen, and gradually disappear after several hours' exposure to the common temperature of the air. In acting on organic liquids, these spots are especially liable to be produced if the flame is weak, and it be kept some time in contact with the porcelain. They are likewise formed in certain cases in simply employing water, zinc, and some kinds of *distilled* sulphuric acid." (p. 412.)

He goes on to remark that they dissolve in strong nitric acid, but not so rapidly as those of arsenic; the residue obtained is yellow or white, and after cooling, on treating this with nitrate of silver, no brick-red arseniate results. He then continues:

"It is impossible to declare that these stains are arsenical, since they do not present all the necessary characters. What is their nature? It is difficult to say. They are probably composed of organic matter and a very minute quantity of arsenic, not susceptible of detection by the usual processes. It may be remarked, however, that about twenty real arsenical stains, of the size of those just mentioned, will yield with nitric acid and nitrate of silver, a brick-red precipitate. We cannot be too cautious when we are required to decide upon the nature of the stains obtained. A witness is not justified in pronouncing them to be arsenical, unless he has found them to possess the first three characters which I have assigned to them." (p. 412.)

We cannot fail to remark here a slight inconsistency, which is sufficient to show us that the author's mind is by no means made up as to the degree of reliance to be placed upon evidence of this kind. The stains to which he alludes in the first part of the preceding extract, and which he admits occur when there is no evidence of the presence of arsenic, actually possess, although in an imperfect degree, the three characters which he deems all-sufficient in the hands of an experienced man, to determine their real nature. It is true that nothing is said about metallic brilliancy, but those obtained from arsenic, as it is well known, do not always possess this. They are white and opaque, immediately volatilized by heat, are dissolved by nitric acid, and leave a white or yellowish residue on evaporation. The non-production of a brick-red precipitate on adding nitrate of silver, which he does not deem an essential character, is then here the only criterion for an opinion; and the fallacies to which this criterion is exposed, when we are operating on such minute quantities, need not be further adverted to. There is obviously no boundary line to be drawn between stains of this description and those actually resulting from arsenic. The author substantially admits this, in confessing that he is unable to form an opinion of their real nature.

M. Orfila then refers to the case of Soufflard, the first in which arsenic was actually detected in the blood and organs of the human body. (This man destroyed himself by swallowing a large dose of arsenic.) The blood used in the experiment, amounting to about eight ounces, was abstracted from the heart and vena cava. On simply boiling the blood in distilled water, scarcely any was procured; but on incinerating the residue of the clot with nitre, a large proportion was obtained. A parallel experiment on a similar quantity of blood taken from a female patient gave no arsenic, clearly showing that the metal existed in the body of Soufflard, and not in the reagents employed to extract it.

"The upper and lower extremities were boiled in distilled water with potash. The decoction yielded arsenic. A parallel experiment on the lower extremity of an adult, who had not been poisoned, gave no arsenic. The liver, spleen, and lungs of Soufflard treated with nitrate of potash and sulphuric acid were also found to contain the poison; while the same organs of an adult, who had not been poisoned, contained none. It is true that in the last case brown stains resembling those of arsenic were procured; but on examination these were proved to be owing to impurity." (p. 416.)

This case seems to establish clearly that arsenic is absorbed into, and circulated with the blood of a person who has been poisoned by that substance.

The first memoir is terminated with a series of conclusions from the foregoing experiments and observations. We have incorporated the greater number of these in our remarks, and we shall now, therefore, only briefly extract what may be novel or interesting.

"Arsenic is to be found in the urine, as well as in the blood and viscera of those who have taken it. When it cannot be detected in the blood it may be in the viscera and urine, and when it begins to disappear from the viscera, it will be more abundantly found in the urine. It must not be supposed, however, that it is rapidly eliminated from the blood and viscera. In two cases in the human subject it was found in the blood fourteen hours after the ingestion of the poison, in such quantity as to render it probable that evidence of its presence in that liquid would have been obtainable twenty-five and thirty hours after the poison had been taken. The viscera will retain the poison for several days." (p. 418.)

From what has just been said, it is obvious a time must arrive when none will exist in the body, if the individual survives long enough. Hence it is necessary to reserve for analysis the urine of those who are supposed to have taken this poison. With respect to the blood, a few ounces are sufficient for determining the presence or absence of arsenic. Venesection will thus enable us to determine the fact of poisoning, in a person who survives the effects of arsenic. Orfila thinks that venesection should form a part of the treatment, since it not merely allays inflammation, but actually withdraws a portion of the poison from the circulating system. (p. 419.)

The following case, which occurs in the third memoir, will serve to illustrate the preceding remarks:

"Madame N., with the intention of destroying herself, swallowed a spoonful of finely-powdered arsenic. In twelve hours the symptoms were such as to render it necessary to bleed her. Venesection was performed and leeches were applied to the epigastrium. The next day M. Casimir Broussais sent me about ten ounces of blood.........The liquid obtained from the incinerated residue of this quantity when introduced into Marsh's apparatus, gave about twenty small arsenical stains, not much coloured, but brilliant. The patient was greatly relieved by the bleeding, and ultimately recovered." (p. 464.)

This is a case of singular interest. It will serve as a good precedent for medical jurists when they are anxious to obtain evidence of arsenical poisoning in the living subject.

2. The Second Memoir is devoted exclusively to an examination of the means by which the purity of the reagents employed in the process may be satisfactorily determined. It must strike every professional reader, and of course it would occur to an acute barrister, that in these refined chemical experiments the operator is much exposed to error, from the fact of arsenic being accidentally present in the tests or in the apparatus employed. When a witness infers the presence of the poison from the discovery of such minute traces, he is bound to show that no fallacy of this kind could possibly have existed. It is not our intention to follow the author into the minute instructions which he gives on this subject. Sulphuric acid occasionally contains traces of arsenic, but we have tried many specimens of the kind commonly used by chemists in this country without meeting with one possessing this impurity. Neither nitric acid nor nitrate of potash is in general found to contain arsenic, but the zinc of commerce occasionally presents traces of it. We have never met with arsenic in using good laminated zinc; and the impurity is so rare, that Orfila only found it three times in making five hundred trials. Orfila does not appear to be aware of the fact, first noticed, we believe, by Dr. Geoghegan of Dublin, that arsenic is apt to attach itself to the zinc employed when it has been frequently used, and hence the necessity for renewing the zinc in cases of great delicacy. No one, however, acquainted with Marsh's test, would proceed to analyze a liquid by it, until he had first satisfied himself that the materials employed were entirely free from traces of the poison. One or two negative results from the apparatus and materials are not sufficient to establish this. Many must be made in a case of importance, and at a certain interval from each other. We agree with the author, that the vessels of copper, iron, or porcelain, employed for making the decoctions of animal matter, are not likely to yield a particle of arsenic under the circumstances.

There is one point connected with the impurity of tests which M. Orfila does not appear to us to make sufficiently clear. It is well known, and he admits the fact, that no evidence of the presence of arsenic may be obtained when we operate on one organ, while, by taking a larger portion of the viscera, the metal will be procured. No application of this important fact is made in the directions given for trying the purity of the tests, while it is obvious that it must apply with as much force to them as to the organs of the human body. Thus an ounce of nitrate of potash may yield no arsenic, while, by operating on several pounds, *traces* of the poison might be procured. In Orfila's process, large quantities of nitric and sulphuric acid are used, and when by it only the *faintest traces* of arsenic are procured, we must insist upon the necessity of examining for that poison so much of the tests as may have been actually employed in the investigation. Nothing short of this will furnish satisfactory medico-legal evidence of the real source of the poison.

3. The Third Memoir is perhaps the most important of the whole, since it is here we find the latest improvements in the author's analytical process. Many experiments were performed to determine whether nitric acid was preferable to nitrate of potash in decomposing organic matter, as also the degree of heat and the length of time most favorable to the process of incineration. We subjoin his conclusions, which appear to us somewhat confused:

"1. In using nitric acid for detecting arsenic which has been absorbed, we must avoid carbonizing the soft parts rapidly and with flame, or slowly and with the extrication of a pyrogeneous odour; for in either case, the greater part, if not the whole of the arsenic, will be lost.

"2. The loss of arsenic is great in proportion to the quantity of organic matter incinerated.

"3. If we wish to extract all the arsenic present, this will be best accomplished by incinerating the organs in an instant, without flame or ignition, and with the production of a large quantity of thick smoke, and of a bulky and spongy charcoal.

"4. That the organs, in order to undergo this form of carbonization, must be previously dried, and then digested with a fixed quantity of nitric acid, which however varies with each organ.

"5. That this process (with nitric acid) is preferable to that with nitrate of potash, since there is no perceptible loss of metallic arsenic." (p. 454.)

Our space will not allow us to enter into all the details relative to the author's process, but as no complete account of it has yet appeared in English, and it undoubtedly possesses advantages over all the methods hitherto recommended for detecting arsenic in complex organic substances, we feel it a duty to give such a description, as to render it easy to be followed by our readers.

The viscera cut into small pieces are to be boiled for about six hours in a porcelain vessel with distilled water, holding a few grains of pure potash dissolved. This aqueous decoction is filtered, acidulated by muriatic acid, and a current of sulphuretted hydrogen gas is then passed into it. If, instead of the viscera, we employ the upper and lower extremities for making the decoction, the fat must be separated by allowing the liquid to cool before the gas is passed in. After a longer or shorter period, sometimes several days, the yellow sulphuret of arsenic, mixed with animal matter, falls down. The precipitate, whatever may be its nature, is separated by filtration. The filtered liquid, although no longer affected by sulphuretted hydrogen, may still contain arsenic : to prove this, evaporate to dryness, and digest the residue in strong nitric acid, in a way to be presently described. The solid residue of the viscera, left after making the decoction in water, is to be perfectly dried, and then immediately digested in strong nitric acid. In general the decoction in water will have removed the whole of the arsenical compound ; but this subsequent treatment with nitric acid will ensure the entire extraction of any that may remain. Our readers will remark that, in speaking of Soufflard's case, the author stated that, by decoction in water, but little arsenic was obtained from the blood, the greater part being left in the solid residue. This discrepancy leaves us in uncertainty as to what is really the fact.

We will now resume the analysis of the precipitate supposed to contain sesquisulphuret of arsenic. This is of a yellow brown or gray colour : it is to be well washed with distilled water, and then digested several times in a weak solution of ammonia. The alkali dissolves out the sulphuret of arsenic, which is then precipitated by the addition of nitric acid. The sulphuret after this is of a brighter yellow, from having lost a portion of organic matter. It is allowed to subside in a porcelain capsule, and the supernatant liquid is withdrawn from it by means of a pipette. The sulphuret is now dried without removal : one portion of it may be reduced as usual by black flux, another portion may be reserved for decomposition in Marsh's apparatus. To effect this, the sulphuret must undergo a further change before its introduction, since nascent hydrogen cannot remove arsenic from the pure sesquisulphuret. A portion of the yellow powder is heated for a few moments with strong nitric acid, any organic matter present is thereby oxidized and destroyed; the sulphur is transformed to sulphuric, and the arsenic to *arsenic acid*. The surplus nitric and sulphuric acids are expelled by evaporating the liquid to dryness, and the dried residue (arsenic acid) is dissolved in distilled water, and placed in the apparatus, when the evidence of the presence of arsenic is speedily obtained. (pp. 398, 456.)

There is here a very great improvement on the common method. Organic matter, mixed with the sulphuret, has hitherto much embarrassed the experimentalist in his attempts to reduce the metal, and no means of separating it have been devised equal to those above recommended by Orfila. The arsenic acid, it is to be observed, will yield a sublimate with black flux, much more readily than the pure sesquisulphuret.

The author avows that, in his view, the whole of these proceedings are not absolutely necessary; and if he recommends the formation of a decoction by boiling the animal matter, and the use of sulphuretted hydrogen, it is to bow to the general feeling of the profession, by applying an additional test. So far from being necessary, he seldom resorts to it, but proceeds to act at once on the dried organ by nitric acid; or, if the soft parts be abundant, on an aqueous decoction of them evaporated to dryness. (p. 456.)

Different proportions of nitric acid are recommended for operating on different organs,—for these we must refer to the work itself. The proportions appear to us large; thus, for a dried liver weighing twelve ounces, no less than *thirty-four* ounces of strong nitric acid are required. (p. 457.)

The operation with nitric acid is very simple. The whole of the acid is placed in a porcelain vessel, and heated over a slow fire; the animal matter thoroughly dried, and reduced to a fine powder, or to fragments, is gradually added at short intervals. Deutoxide of nitrogen is abundantly evolved, and the animal matter dissolves. If much of this be put in at once, or it be not thoroughly dried when introduced, a considerable quantity of froth is formed, which by boiling over may cause a loss of arsenic. The liquid, which is at first yellow, becomes orange-coloured, and finally of a deep red; it is at the same time inspissated by evaporation. In continuing the heat, the whole of the matter becomes carbonized; and when a thick black smoke begins to escape, the vessel is to be removed from the fire. The whole of the arsenic is here converted to arsenic acid, and this is easily removed from the carbonaceous residue by digesting it in distilled water. The solution is, if necessary, concentrated by evaporation, and then introduced into Marsh's apparatus, when the evidence of arsenic will be obtained. If the whole body is to be examined, separate decoctions of the muscles and organs may be made, these evaporated to dryness, and the dried residues united and digested in nitric acid in the manner described. (p. 458.)

The author has found some inconvenience from the froth produced on introducing the solution of arsenic acid into Marsh's apparatus. He therefore advises the use of a large quantity of olive oil; we should prefer employing alcohol for reasons already stated. At the same time, if the resulting solution of arsenic acid were so viscid as to produce much froth, it appears to us that this might be got rid of by evaporating it to dryness, again digesting in nitric acid, evaporating, and then redissolving in distilled water.

"In these experiments, care must be taken that too much nitric acid is not employed, that the heat is not too intense; and that, during carbonization, the

LIBRARY OF THE

Orleans Parish Medical Society contents do not burst into flame. Under any of these accidents, the arsenic might be entirely dissipated." (p. 460.)

The efficacy of this process was well tested as to time in the two following cases:

"The liver from the body of N. M., suspected to have died from poison, and which had been interred for five months, was carbonized by nitric acid. The aqueous decoction yielded an abundance of arsenical stains." (p. 463.)

"I obtained from the heart, mesentery, omentum, and a portion of the intestines of M. Cumant, a small quantity of arsenic. This person died in December, 1838: his body was exhumed in July, 1839, and the organs above mentioned were examined in the month of December following." (p. 464.)

When nitrate of potash is used for the process of incineration instead of nitric acid, then a large quantity of sulphuric acid is required to separate the alkali from the resulting carbonate and arseniate. The sulphate of potash formed, is separated by crystallization. The arsenic acid is freed from any excess of sulphuric acid by evaporation; but if Marsh's apparatus be used, this last step is not necessary.

4. The Fourth Memoir describes the discovery of the presence of arsenic, as a natural constituent of the human body. This is a startling novelty, and the evidence upon which the statement is based therefore requires close examination. The author affirms that arsenic exists in human bone, as well as in that of the dog, ox, and sheep. The arsenical compound existing in bone cannot be extracted by boiling that substance in water, hence no medico-legal difficulty can arise on that ground. The arsenic is lost when the bone has been intensely calcined, and when the calcination has taken place in air, or in immediate contact with the heated fuel. The presence of arsenic was detected

"By heating the bones above an intense fire without contact until they become friable and of a grayish-white colour. They were then finely powdered and sifted. *Eight ounces* of the powder were digested in distilled water, with three ounces of sulphuric acid; after four days more distilled water was added, the mixture boiled, and the loss by evaporation made up. The filtered liquid, placed in Marsh's apparatus, yielded numerous brown arsenical stains of great brilliancy and thickness." (p. 467.)

"Arsenic was also detected in bone which had been carbonized in a common crucible, at a high temperature, but without access of air." (*Ibid.*)

In boiling six ounces of common bone ash with distilled water, containing a small quantity of pure potash, a liquid was obtained which, on filtration and neutralization by sulphuric acid, gave arsenical stains with Marsh's apparatus. (p. 471.) We are at a loss to reconcile this statement with the precautions which the author had just before laid down as absolutely necessary for determining the presence of arsenic in bone. We have tried the bone ash of commerce without detecting any trace of arsenic.

When we look to the number of eminent chemists who have analyzed bone without finding any arsenic present, we are inclined to think that the experiments of M. Orfila require corroboration, before we admit the existence of arsenic in that substance as a natural constituent. His enthusiastic devotion to the subject may have led him to overlook some source of error. Besides, he appears to have relied upon the use of Marsh's apparatus alone, whereas, did arsenic exist so abundantly as his results would lead us to suppose, other corroborative tests might be employed. May not the deposit have been some combination of phosphorus? At any rate we must remind him, when he relies so exclusively on one test, of what he is reported to have said on the recent trial of Madame Laffarge. "Marsh's apparatus is of recent date. It has not yet been perfectly studied by every one, and even those who have studied it frequently find themselves embarrassed in using it."

His observations on this subject require revision. Thus he has not settled whether the alleged arsenic exists in the animal or in the earthy part of bone, nor has he analyzed bone in the humid way, that is, in separating the animal matter by dilute muriatic acid. The arsenic in this case should be found in the acid menstruum, or in the separated gelatin.

In submitting the blood and viscera of subjects which had not been poisoned to these analytical processes, he has not been able to procure the smallest evidence of the presence of arsenic in them. At the same time he will not undertake to say positively that it is absent. The present means for its detection may not be sufficiently delicate; a more improved process, or the bare fact of operating at once on fifteen or twenty brains, or a like number of livers and lungs, might lead to the discovery of the metal as a constituent even of these viscera. M. Orfila might have added that by operating on subjects of different ages, different sexes, and different states of health, arsenic might in some instances be discovered, at least to the same extent that it exists in bone. This we consider to be the height of transcendental analysis. M. Orfila seems to be little aware from the uncertainty in which he leaves the subject, that he is giving the benefit of an *argumentum ex concesso* to those who are opposed to his views. To our minds it is left uncertain whether by more numerous experiments on different subjects he might not obtain the same evidence of the existence of arsenic in the blood and viscera, as in the skeletons of healthy subjects; and thus, all the practical benefit to science, derivable from his experiments, would be frittered away.

CHARITY HOSPITAL, MEDICAL LIBRARY.

We have yet purposely avoided speaking of the *muscular* system. Do the muscles of the human body contain arsenic or not? M. Orfila declines answering the question one way or the other; all that he says is: "*It is not proved that they do.*" (p. 476.) Many experiments were performed by him to determine this point; and as a general result, he admits that he obtained from healthy muscle, treated by his process, white, opaque and volatile stains, some brown and dull, others small and brilliant, or as we understand it, "metallic." (pp. 476-81.) These stains are said to be not very soluble in cold nitric acid; but dissolved by it when diluted and boiling, leaving a white residue on evaporation. The author acknowledges that the stains may differ a little in character, owing to the arsenic (?) being in very small quantity, but he observed: "If I do not affirm that muscle contains arsenic, I will not conclude from my experiments that it does not contain any." (p. 482.) From his facts we feel bound to conclude, either that the rules by which he has determined the presence of minute traces of arsenic in other cases are erroneous, or that human muscle really does contain that poison as a natural constituent. With a larger quantity of muscle than he employed, or with an improved process, he thinks arsenic might be demonstrated to exist in the muscular system. (*Ib.*)

Although he contends that the stains from muscle may be distinguished from those which are truly arsenical, yet he advises a witness in searching for arsenic in a suspected case, to confine his analysis to the viscera. "He may thus avoid objections, which, although of little value, might still have an influence on the minds of certain juries." (!) (p. 485.)

The author appears to us to leave this part of his subject in great obscurity. At p. 476 seven experiments are related, in which stains resembling those of arsenic were procured in each case from muscle, although not obtained from poisoned subjects. At p. 416 we find that the production of similar stains from muscle in Soufflard's case, was taken as evidence of poison having been absorbed, and conjointly with this, the lower extremity of a normal subject was submitted to the same operation without any stains resulting! How are such differences to be reconciled? It seems to us clear from the experiments (p. 476) that the production of stains from muscle is no evidence of poisoning; while their non-production in the parallel experiment connected with Soufflard's case, is wholly opposed to the results of the seven experiments subsequently detailed.

The Fifth Memoir involves many new points which are likely, if corroborated, to give rise to some medico-legal discussion. The first question which the author proposes to examine is, whether arsenic ever exists in the soil of cemeteries or graveyards. He answers this in the affirmative; but the admission is confined to those cases in which the earth is greatly mixed with the fragments of bones. Indeed, it is his opinion that the arsenic is simply that which he alleges to be naturally contained in bone. It was not sufficient in these cases to digest the soil in hot or cold water and operate on the decoction. He found it necessary to macerate the earth for several days in dilute sulphuric acid, and then boil the whole for some hours in strong sulphuric acid. (p. 493.) Even in these cases the stains obtained, were extremely small, and the arsenic only in infinitesimal quantity. Assuming that arsenic really exists in the earth of cemeteries, we find many experiments performed for the purpose of ascertaining whether in such a case it is liable to be infused into a body by the draining of water, so as to give the idea of the person having died from poison. Without actually denying that this might occur, the author thinks it "difficult to admit that any soluble combination of arsenic could so penetrate into a body as to give the idea of poisoning: nevertheless, a serious mistake might be made, unless, before proceeding to the analysis, all the arsenical earth was removed from the exterior." (p. 499.) Then we find him suggesting that cases of this sort might be detected by making separate analyses of the exterior and interior of the body, and noting the relative proportions of arsenic in each!

Lastly, Is the body of a poisoned individual likely to lose the arsenic present in it, by the drainage of water after long interment. This question relates not so much to the poison existing in a free state in the viscera, as to that minute portion which has become absorbed into and incorporated with them. There are no facts to decide it; but the author thinks it very unlikely that this small portion of poison could be thus removed from the body, so long as any visible traces of members or viscera remain. (p. 503.)

Among the extraordinary conclusions in this memoir, we may notice the following:

"If in the soil of a cemetery we discover an arsenical compound, *soluble in cold water,* there would be strong reason to presume that it came from some bodies in the neighbourhood, *unless it were proved that that part of the cemetery had been watered with a solution of arsenious acid, or some other arsenical preparation.*" (p. 504.)

We have placed the last lines in italics to show how far the author deviates from what is practical, and how prone he is to work out his argument *ad infinitum.* It appears to us that it would have been only consistent in him to have taken into consideration the possible presence of arsenic in the iron or brass materials used about a coffin, and to have made further exceptions on this ground. Seriously, however, we must declare our conviction that these speculations are liable to do mischief instead of benefiting the science of medical jurisprudence. We know that the latter is really the author's object, and that the science owes him much; but we are sorry to find him advising that researches so crude and ill-digested should ever be made the basis of medical evidence.

All the reasoning in the last memoir depends on the admission of the presence of arsenic as a constituent of bone; but we do not think that this point is yet satisfactorily established. We allow that the author may have obtained stains resembling those of arsenic, not only from bone but from the soil of cemeteries: still we learn from his own statements that unless these stains be of a very decided character, there is no possibility of distinguishing them from those which are not arsenical. We think he is too much disposed to rely upon slight appearances, and to take what is barely probable for what is real: this is the only way in which we can explain several inconsistencies into which he has been led in these memoirs. We must here remind him of his own observation on a late memorable occasion in cases of poisoning. "Medical jurisprudence is not satisfied with suppositions, it requires positive proofs. The metal must be reproduced"—we would add—to the reasonable satisfaction of all chemists.

In these memoirs there are many sound practical remarks: the process for extracting arsenic from organic mixtures is the best in our judgment which has been hitherto devised. We could have wished to see some of the author's opinions based upon less questionable grounds, since they are likely to be extensively circulated, and have much influence on the practice of medical jurisprudence.

The Sixth Memoir refers to some facts connected with *tartarized antimony* as a poison. These are new and therefore require to be briefly adverted to. Antimony, as we have already seen, is susceptible of being reduced by hydrogen in Marsh's apparatus. Very soon after Mr. Thompson's discovery of this fact, the apparatus was used in this country as a test for antimony; but M. Orfila, has, we believe, the credit of first employing it to detect the presence of that metal in the organs of the body. Magendie had inferred from his physiological experiments that it was actually absorbed into the circulation, but there were no means of demonstrating its positive existence in the blood and secretions. The author having satisfied himself that antimony might be detected in organic mixtures by a process analogous to that which he had successfully employed for arsenic, proceeded to determine the question by chemical analysis.

As general results he found, "1, that tartarized antimony introduced into the stomach or applied to the cellular tissue is quickly carried into the substance of the viscera, where it remains but a short time, especially in the non-secretory organs; 2, that after having left these viscera, it is eliminated with the urine and probably also with the other secretions." (p. 517.)

We learn further that from the well-known emetic powers of this body it is likely to be soon expelled from the stomach; and chemical analysis may fail to detect it in that organ, when it will still be found in the other viscera, which it may have reached by absorption. The liver and the kidneys generally retain traces of the metal longer than the other organs, owing to the blood remaining longer in them. The salt appears to be only partially decomposed; for it is capable of being, to a certain extent, removed from the blood and soft parts by boiling water; but the process for its complete extraction is a little more complex. The viscera being thoroughly dried are to be decomposed by nitric acid, as in the case of arsenic. The resulting carbonaceous residue is to be boiled in muriatic acid with a few drops of nitric acid, the liquid filtered, and then introduced into Marsh's apparatus. In this case, the salt of antimony present is reduced, and chloride of antimony is formed by digesting the residue in nitro-muriatic acid. Should we be examining a liquid, we have only to evaporate to dryness, and proceed with the residue in the same way. The author finds that the metal may be discovered in the urine when it has left every other organ and secretion; but under all circumstances, it disappears from the body with much greater rapidity than arsenic.

The metallic stains suspected to be antimonial are to be identified by the means already pointed out.

In the Seventh and last Memoir we have some account of the salts of *copper*, and the method of detecting that metal in the viscera of the body. Before attempting to determine whether or not, in cases of poisoning, the metal copper was conveyed into the circulation, it was of course necessary to find out a sure and delicate process for detecting its presence. Copper being a fixed metal, a very different proceeding was here required to that adopted in the case of arsenic and antimony. After many trials, Orfila came to the conclusion, generally agreed upon among chemists, that the deposit of the metal on iron is the best test, when it exists only in minute traces.

The salts employed in the experiments were the acetate and the sulphate; and the object was to ascertain whether any portion of the metal had become absorbed and carried into the viscera. Dogs were selected for the experiments: in some cases the salts were introduced into the stomach, and the œsophagus tied; in others, the powdered salt was placed in a wound made in the skin on the inside of the thigh. The animals either died from the effects of the poison or were killed, and their viscera immediately examined.

The following plan was resorted to for detecting copper. The viscera were cut into pieces and boiled for six hours in a quantity of distilled water acidulated with a few drops of nitric acid. The decoction was filtered and evaporated to dryness. The dry residue was carbonized by heating it in strong nitric acid; and the carbonaceous ash was then treated with muriatic, mixed with a few drops of nitric acid. This was filtered and again evaporated to dryness; the residue was then dissolved in water acidulated with sulphuric acid. On introducing a piece of polished iron into this liquid, a certain quantity of copper was deposited on it in the course of an hour. (p. 533.) Many hours are sometimes required for this deposit to take place, where the quantity of metal is small.

In this way, copper was detected in the lungs, heart, liver, spleen, and kidneys of animals experimented on; nor did the results vary, whether the analysis were performed on the organs extracted from the recently killed animal, or from one which had been some time dead. It is worthy of remark, that there was no satisfactory evidence of copper either in the blood or in the urine of these animals. (pp. 540-2.) The experiments, however, clearly show that the metal in some form or other does find its way into the circulation.

But since copper is said to be a natural constituent of the healthy animal organs, the author deemed it necessary to perform some experiments in order to enable medical witnesses to distinguish this normal copper from that introduced into the system as a poison.

In operating on the viscera of the healthy human body and dogs, in the way already described, not a trace of that metal could be found. Water, especially at a boiling temperature, will dissolve the greater part of the copper introduced into the viscera as a poison; but it will not remove any portion of that which is a normal constituent of the organ. This normal copper can only be detected by drying and incinerating the greater number of the viscera of the *human* body, and then it is only found in very minute traces. The same organs in the healthy dog, similarly treated, do not yield any portion of the metal. This difference is ascribed by Orfila not to the entire absence of copper from the viscera of the dog, but to the fact that they occupy much less bulk than in the human subject, and therefore that the proportion of copper is probably too small to be detected by the same chemical process. (p. 549.)

We do not consider it necessary to enter into the question, relative to the transudation of the poison through the alimentary canal into the surrounding viscera. A question of this kind does not appear to us to have any practical bearing. The fact of imbibition taking place after death is well known. The only possible case in which any doubt could arise from such a circumstance is in the very improbable occurrence of the introduction of copper into the stomach of a dead person. Here imbibition might give rise to the idea of the poison having been circulated after absorption; but as, in such a case, we should have first to account for the presence of a salt of copper in the stomach, the minor would merge in the major question.

With this we conclude our notice of M. Orfila's late discoveries. Many of these are in a medico-legal and physiological view of great value and practical importance. We have already expressed our opinion of the Memoirs on Arsenic. The author has there shadowed out some lines of investigation, which we hope he will pursue with the same zeal which he has hitherto shown. The Essays on Antimony and Copper will be rendered more complete by his extending his experiments, whenever an opportunity occurs, to the human subject. Neither antimony nor copper has yet been found in the blood of man or animals poisoned by the salts of these metals. Antimony has been discovered in the urine of persons who had taken large medicinal doses of this substance, but copper has not been detected under these circumstances, in any of the human organs or secretions. However valuable the results of experiments on animals may be to the physiologist, observations on man are of infinitely greater value to the medical jurist.

---

